# Association of a Novel Prognosis Model with Tumor Mutation Burden and Tumor-Infiltrating Immune Cells in Thyroid Carcinoma

**DOI:** 10.3389/fgene.2021.744304

**Published:** 2021-12-17

**Authors:** Siqin Zhang, Shaoyong Chen, Yuchen Wang, Yuxiang Zhan, Jiarui Li, Xiaolin Nong, Biyun Gao

**Affiliations:** ^1^ College of Stomatology, Guangxi Medical University, Nanning, China; ^2^ Guangxi Key Laboratory of Oral and Maxillofacial Surgery Disease Treatment, Nanning, China

**Keywords:** tumor mutational burden, tumor-infiltrating immune cells, immunotherapy, prognosis, thyroid carcinoma

## Abstract

Although immunotherapy has recently demonstrated a substantial promise in treating advanced thyroid carcinoma (THCA), it is not appropriate for all THCA patients. As a result, this study aims to identify biomarkers for predicting immunotherapy efficacy and prognosis in THCA patients based on a constructed prognostic model. The transcriptomic and corresponding clinical data of THCA patients were obtained from the Cancer Genome Atlas (TCGA) database. We identified differentially expressed genes (DEGs) between THCA and normal samples and performed an intersection analysis of DEGs with immune-related genes (IRGs) downloaded from the ImmPort database. Functional enrichment analysis was performed on the chosen immune-related DEGs. Subsequently, Cox and LASSO regression analyses were conducted to obtain three hub immune-related DEGs, including PPBP, SEMA6B, and GCGR. Following that, a prognostic risk model was established and validated based on PPBP, SEMA6B, and GCGR genes to predict immunotherapy efficacy and THCA prognosis. Finally, we investigated the association between the constructed risk model and tumor mutational burden (TMB), abundance of tumor-infiltrating immune cells (TICs) as well as immunotherapeutic targets (PDL-1, PD-1, and CTLA4) in THCA. THCA patients in the high-risk score (RS) group showed higher TMB levels and worse prognosis than the low RS group. Patients in the high-RS group had higher proportions of monocytes, M2 macrophages, and activated dendritic cells, whereas those in the low-RS group exhibited higher numbers of M1 macrophages and dendritic resting cells. Our data implied that the constructed THCA prognostic model was sound and we concluded that the THCA patients having high TMB and low PD-L1 expression levels might respond poorly to immunotherapy. Taken together, we constructed a novel prognostic model for THCA patients to predict their prognosis and immunotherapy efficacy, providing a viable option for the future management of THCA patients in the clinic.

## Introduction

Thyroid carcinoma (THCA) is the fifth most prevalent malignancy affecting women (Zhang et al., 2019b) and a major cause of annual endocrine malignancies death (Aboelnaga and Ahmed., 2015). Over the past decades, the incidence of THCA has been escalating worldwide (Cabanillas et al., 2016). THCA is generally classified into four pathological subtypes: papillary thyroid cancer (PTC, 80–85%), follicular thyroid cancer (FTC, 10–15%), medullary thyroid carcinoma (MTC, less than 2%) and anaplastic thyroid carcinoma (ATC, less than 2%) (Laha et al., 2020). Although most THCA is well-differentiated PTC with a 10-years survival rate of over 95%, some variants of PTC may demonstrate increased aggressive behavior, particularly in older patients, contributing to significant mortality (Nath and Erickson., 2018). Therefore, surgical resection and radioiodine (RAI) therapy were considered standard treatments for most THCA patients. Nonetheless, survival rates for advanced thyroid cancer patients remain low. For these reasons, novel therapeutic strategies, such as immunotherapies and targeted molecular therapy, are under investigation for treating advanced or metastatic THCA patients.

In recent years, immune checkpoint inhibitor (ICI) has achieved remarkable progress in treating breast cancer (Santa-Maria and Nanda., 2018), lung cancer (Anagnostou et al., 2017), melanoma (Amaria et al., 2018; Barrios et al., 2020) and squamous cell carcinoma of the head and neck (HNSCC) (Ferris et al., 2016; Ferris et al., 2018). It has been shown that numerous immune cells and their mediators exist in the tumor microenvironment (TME) of THCA with various interactions between them (Varricchi et al., 2019). In addition, it was demonstrated that increased frequency of regulatory and PD-1^+^ T cells was associated with the recurrent or aggressive PTC (French et al., 2012), and that the frequency of PD-1^+^ T cells was higher in patients with extranodal invasion than in those without lymph nodes metastases, indicating that it may be associated with the THCA prognosis. Furthermore, Gunda and his colleagues have recently revealed that anti-PD-1/PD-L1 therapy could beneficially modulate the immune microenvironment in orthotopic ATC murine model while simultaneously enhancing the efficacy of lenvatinib, a multi-targeted tyrosine kinase inhibitor (Gunda et al., 2019). The efficacy of ICIs has also yielded encouraging results in clinical trials, manifesting potent antitumor effects and improved tolerability in advanced FTC patients (Mehnert et al., 2019). However, the expensive cost of ICIs, which averages around $150,000 per year (Oiseth and Aziz., 2017), and their unpredictable efficacies (Giannone et al., 2020), prelude their widespread clinical use. Considering these factors, screening for appropriate molecular markers to improve treatment precision is highly demanding.

Tumor mutational burden (TMB), or the number of nonsynonymous mutations in a genomic region of somatic cells, is a biomarker employed to predict immunotherapy efficacy in various cancers (Samstein et al., 2019). As is widely known, tumor-infiltrating immune cells (TICs), an essential component of TME, are critical for in tumor initiation and progression (Bissell and Hines., 2011). Furthermore, gene locus mutations are observed in many histological subtypes of THCA, such as anaplastic lymphoma kinase (ALK), neurotrophic receptor tyrosine kinase 1 (NTRK1) genes rearrangements, BRAF and GTPase RAS family genes (Bos, 1989; Nikiforov and Nikiforova., 2011; Cancer Genome Atlas Research Network, 2014; Arndt et al., 2018; Varricchi et al., 2019). As high-throughput sequencing advances, large-scale acquisition of relevant cancer genomic data has become possible. However, few studies have conducted in-depth investigations to evaluate immunotherapy efficacy and prognosis in THCA.

Herein, based on Cancer Genome Atlas (TCGA) and ImmPort databases, we screened immune-related differentially expressed genes (IRDEGs), explored their functional enrichment, and constructed a prognostic prediction model. Additionally, we further investigated the association of TMB with immune infiltration and prognosis in THCA.

## Materials and Methods

### Flow Diagram of Analysis

**FIGURE 1:**
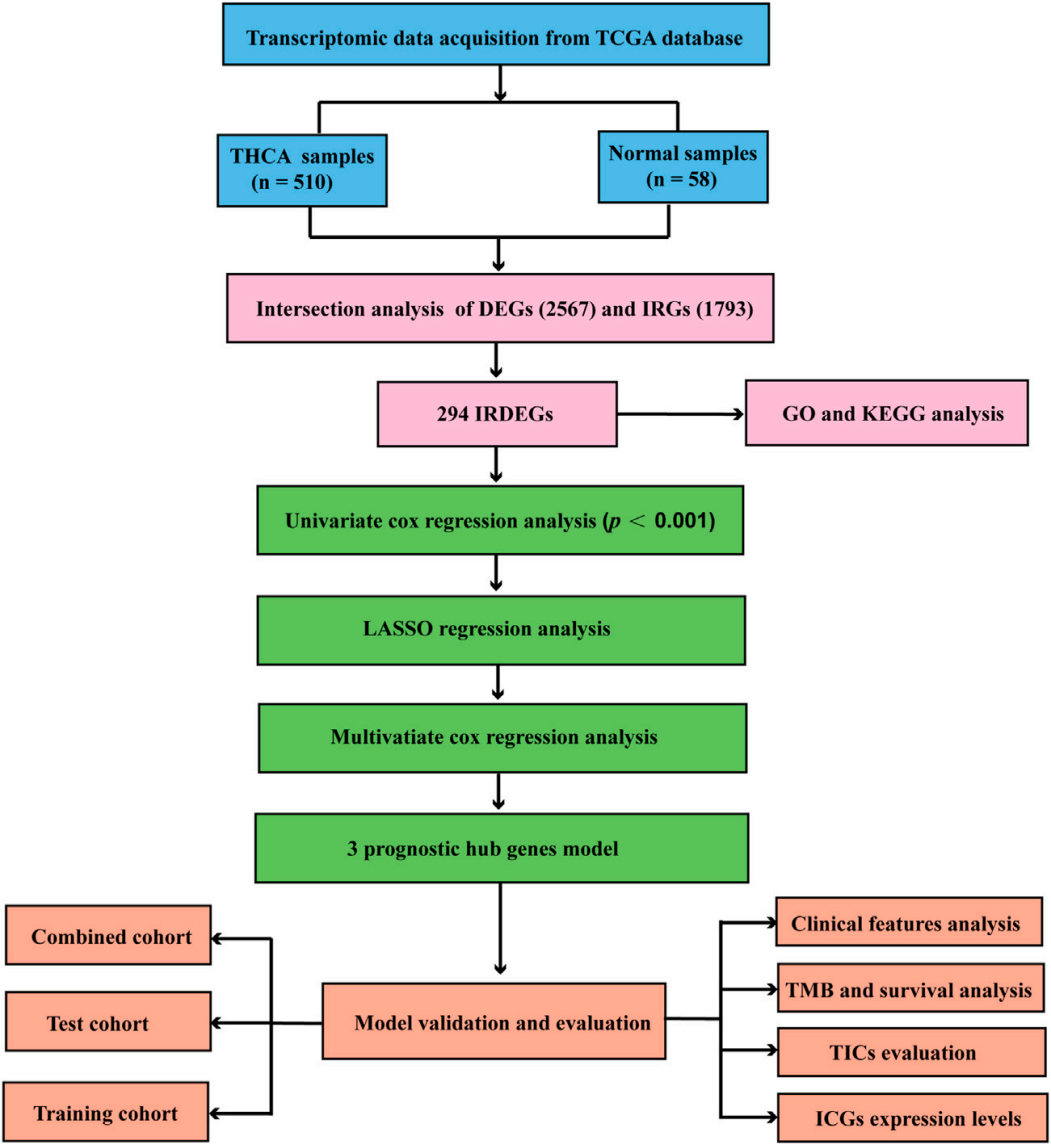
Flow chart for construction and validation the THCA prognostic model. THCA, thyroid carcinoma.

We designed a flow chart of analysis for the construction, validation and evaluation of the prognostic model in THCA. The analysis process was performed strictly according to the flow chart (Figure 1).

### Data Downloading and Processing

We downloaded transcriptomic data with HTSeq-FPKM workflow type from TCGA of THCA project database (https://portal.gdc.cancer.gov/), including 510 tumor and 58 normal samples. Besides, masked somatic mutation data processed by VarScan software was also acquired from the GDC (Genomic Data Commons) data portal of TCGA database. Next, we downloaded clinical data of corresponding patients, including their age, gender, tumor grade, tumor stage, survival time and survival status.

### Screening of Immune-Related Differentially Expressed Genes

Firstly, differentially expressed genes (DEGs) between normal and tumor tissues of THCA were analyzed using R software “limma” package^4^, and the screening criteria were false discovery rate (FDR) < 0.05 and |log2FC| > 1. Immune-related genes (IRGs) were obtained from ImmPort database (http://www.immport.org/) and then performed an intersection analysis on DEGs and IRGs to identify IRDEGs. R “venn” package was used to visualize to result of intersection analysis and R “pheatmap” package was performed to manifest the expression levels of IRDEGs of the THCA samples in the TCGA database.

### Functional Enrichment Analysis

We used R package “org.Hs.eg.db” to obtain Entrez-ID of each IRDEG, and then performed gene ontology (GO) and Kyoto Encyclopedia of Genes and Genomes (KEGG) pathway analyses on IRDEGs using “clusterProfiler,” “enrichplot,” and “ggplot2” R packages. *p* < 0.05 and *q* < 0.05 were utilized as statistical significance thresholds.

### Construction and Validation of a Prediction Model

For cross-validation, we randomly divided all samples into two groups with a 2:1 ratio using R package “caret”, referred to as the training cohort and the test cohort, containing 2/3 and 1/3 of THCA cases, respectively. First, we determined IRDEGs associated with THCA survival in the training cohort using univariate Cox regression analysis with a threshold value of *p* < 0.001 via R package “survival”. Then, key IRDEGs were identified using LASSO regression using R package “glmnet”, and multivariate Cox regression analyses, thus constructing a THCA prediction model. Following that, we divided all THCA samples into high- and low-risk score (RS) groups according to their median RS. For high- and low-RS groups, Kaplan-Meier survival analysis and time-related receiver operating characteristic (ROC) curve analysis through R package “timeROC”, were employed to cross-validate the predictive power of the model in the training test and the combined cohorts (containing all THCA samples).

### Analysis of Tumor-Infiltrating Immune Cells

We utilized CIBERSORT algorithm (R script v 1.03), a deconvolution algorithm to quantify immune cell proportions based on transcriptomic expression profiles (Newman et al., 2015; Yao et al., 2020), to calculate the relative frequencies of 22 tumor-infiltrating immune cells (TIC) subtypes in tumor samples with high and low RS groups. *p* < 0.05 was utilized as statistical significance thresholds, and Wilcoxon test was performed to compare differences in the relative frequencies of various type of immune cells.

### Analysis of High- and Low-RS Groups in Terms of their Clinical Information

After establishing the prediction model, we conducted the difference analysis to present the diversity of the clinical information between the high- and low-RS groups through R packages “ggpubr” and “limma”. Next, Kaplan-Meier survival analysis was used to compare the survival outcome of patients between the two groups, and performed the difference analysis of TMB levels and immune checkpoint genes (ICGs) expression levels between high- and low-RS groups to predict their treatment responses when receiving immunotherapy.

### Statistical Analysis

Statistical analyses were performed using R language (version 4.0.3). Kaplan-Meier survival analysis was used to assess the differences of the survival outcome between different groups. Univariate and multivariate Cox regression analyses were used to identify independent prognostic factors. *p* value < 0.05 was considered statistically significant in all tests.

## Results

### Identification of DEGs and IRDEGs

**FIGURE 2:**
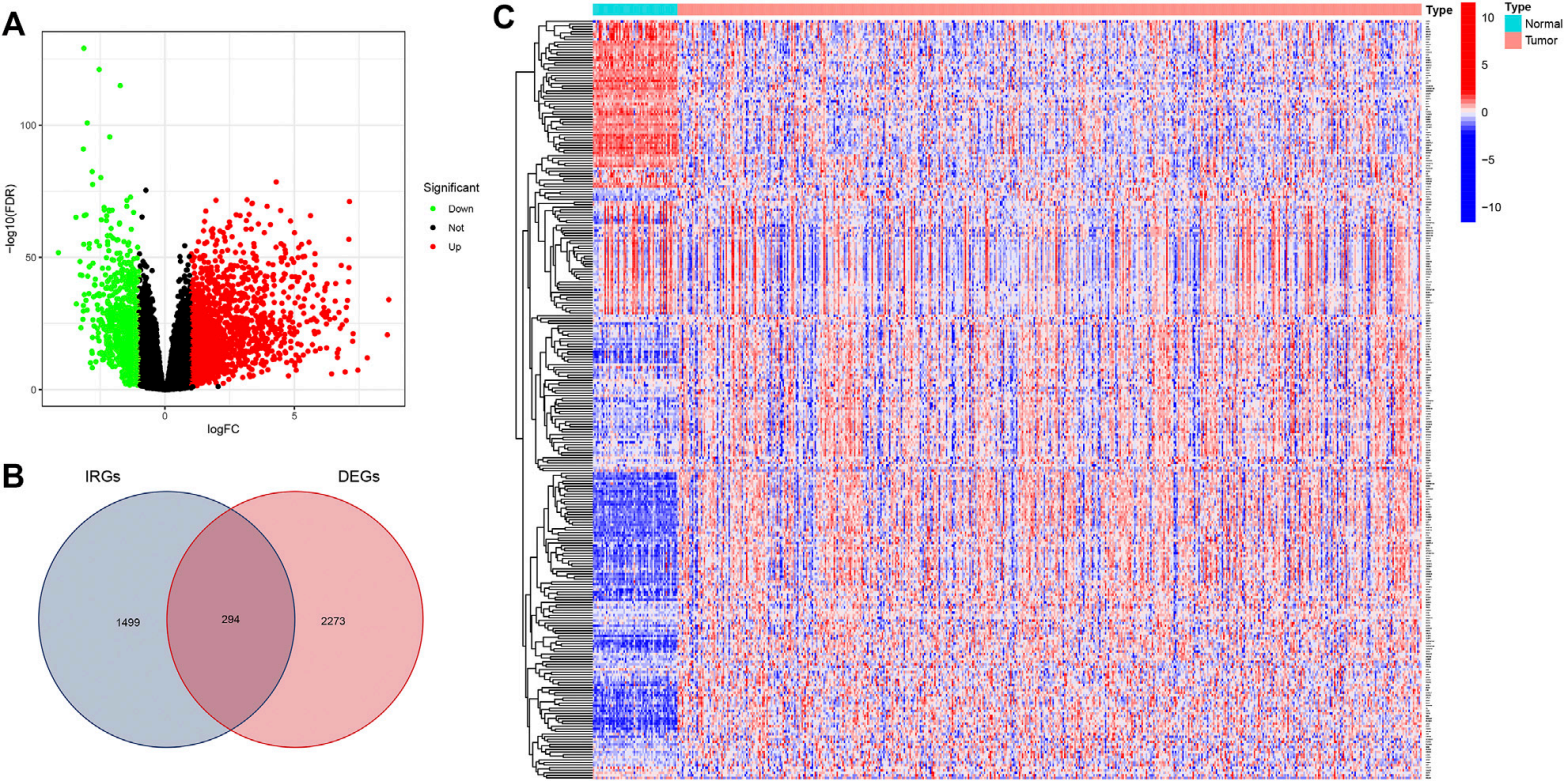
Identification of immune-related DEGs in THCA. **(A)** Volcano plot for DEGs between THCA and normal samples from TCGA database. **(B)** Intersection of 2567 DEGs and 1793 IRGs in Venn plot **(C)** Heatmap showing immune-related IRDEGs. DEGs, differentially expressed genes; IRGs, immune-related genes.

We first used R software “limma” package^4^ with false discovery rate (FDR) < 0.05 and |log2FC| >1 to identify DEGs between normal and THCA tissues, resulting in 2567 DEGs, as displayed in the volcano plot (Figure 2A). Then 294 IRDEGs were obtained from the intersection analysis of 567 DEGs and 1793 IRGs from ImmPort database and displayed in a Venn diagram (Figure 2B). expression levels of 294 IRDEGs between tumor and normal samples were visualized via a heatmap (Figure 2C).

### Functional Enrichment Analysis for IRDEGs

**FIGURE 3:**
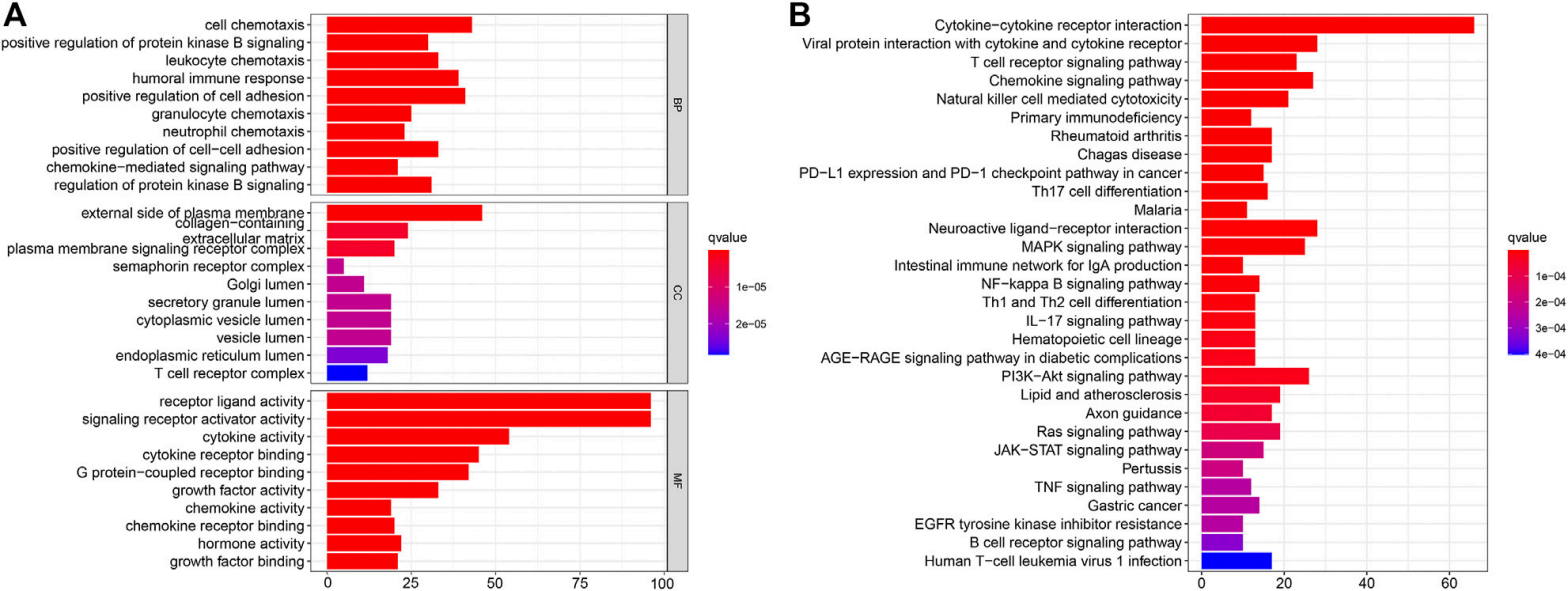
Enrichment analysis of IRDEGs. **(A)** GO enrichment analysis. **(B)** KEGG pathway enrichment analysis. IRDEGs, immune-related differentially expressed gene; GO, gene ontology; KEGG, Kyoto Encyclopedia of Genes and Genomes; BP, biological process; CC, cellular component; MF, molecular function.

To further investigate relevant pathway, biological process, cellular component, and molecular function of 294 IRDEGs, we performed GO and KEGG pathway enrichment analysis for these IRDEGs, as shown in Figure 3. GO enrichment analysis indicated that these IRDEGs were mostly involved in chemotaxis-related activities, cell adhesion regulation and other immune-related responses (Figure 3A). Besides, KEGG pathway analysis revealed that these IRDEGs were chiefly enriched in immune-related pathways, such as cytokine−cytokine receptor interaction, Viral protein with cytokine receptor, T cell receptor signaling pathway, Chemokine signaling pathway, Natural killer cell mediated cytotoxicity, PD−L1 expression and PD−1 checkpoint pathway in cancer (Figure 3B).

### Construction and Validation of THCA Prognosis Model

**FIGURE 4:**
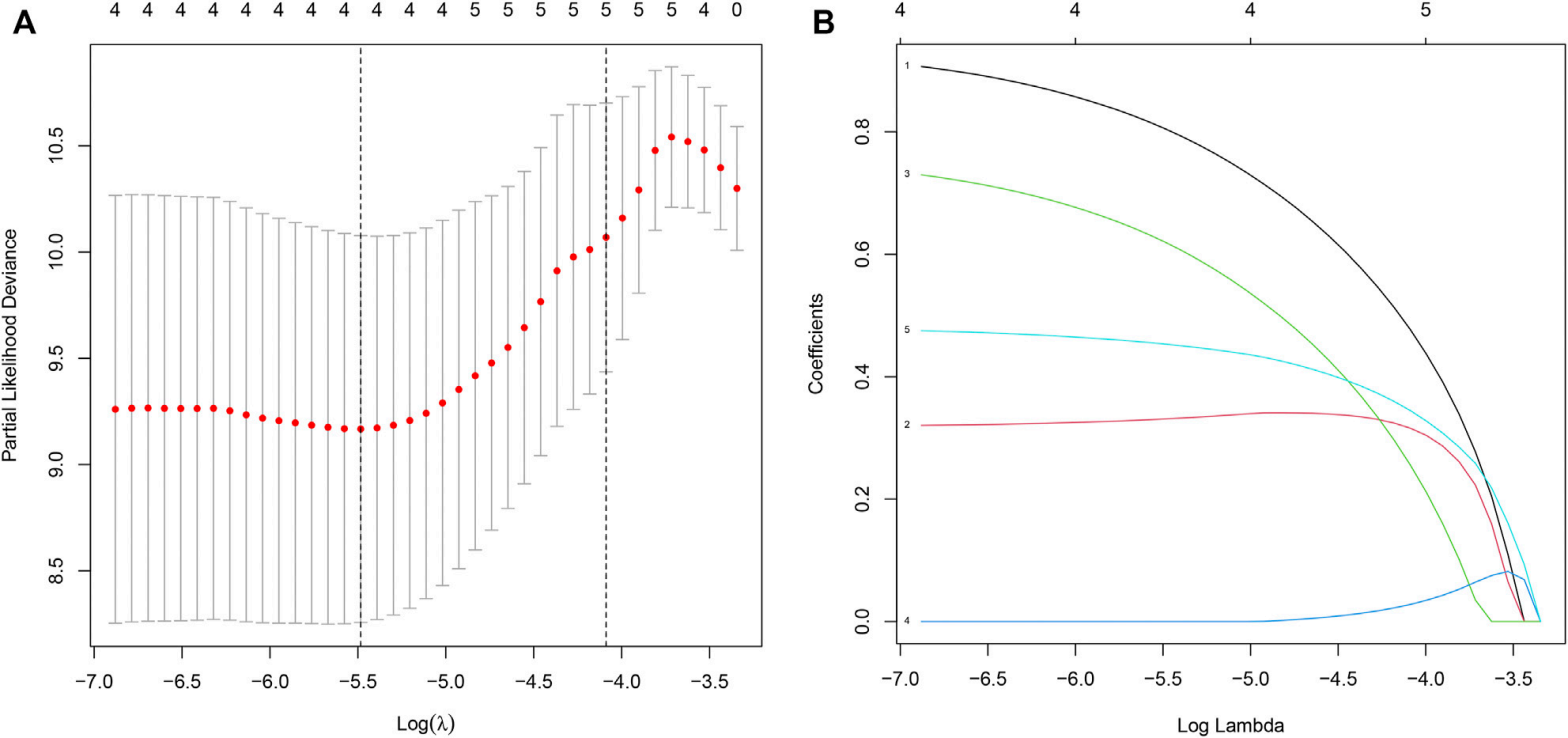
Identification of IRDEGs associated with THCA prognosis. **(A–B)** LASSO coefficient profiles of prognostic-related IRDEGs. LASSO, least absolute shrinkage and selection operator. IRDEGs, immune-related differentially expressed genes.

**FIGURE 5:**
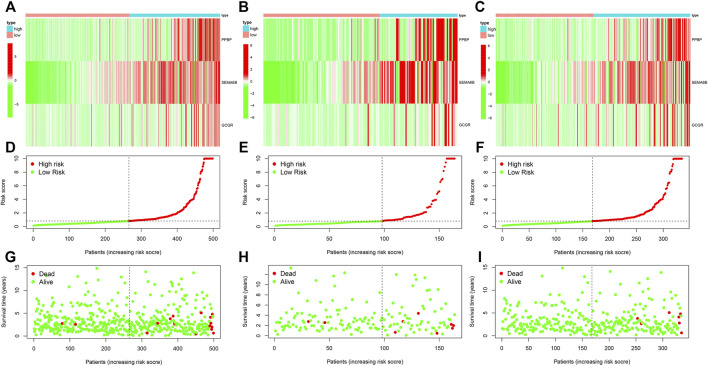
Construction of THCA prognostic model **(A–C)** Expression levels of three prognostic hub IRDEGs among THCA patients in the combined test, and training cohorts, respectively. **(D–F)** The distribution of RS among THCA patients in three cohorts. **(G–I)** The survival status correlated with RS among THCA patients. THCA, thyroid carcinoma; RS, risk score.

We first performed univariate Cox regression analysis on 294 IRDEGs to find the key IRDEGs that affect patients’ survival outcome with a threshold value of *p* < 0.001, which five genes (PPBP, RBP4, SEMA6B, VGF and GCGR) were selected as being significantly associated with THCA patients’ prognosis in this step (Table 1). Subsequently, LASSO regression was used for the following analyses (Figures 4A–B), thereby obtaining four candidate genes (PPBP, RBP4, SEMA6B, and GCGR). Eventually, three prognostic hub genes (PPBP, SEMA6B and GCGR) were screened from five these candidate genes by multivariate Cox regression analysis (Table 1). Next, we randomly assigned all THCA cases into training and test cohorts at a 2: 1 ratio for cross-validation, referred to as the training cohort and the test cohort, containing 2/3 and 1/3 of THCA cases, respectively. Additionally, no significant difference was observed in the clinical characteristics between the training and test cohorts (*p >* 0.01, Table 2). The THCA prognostic model was constructed based on the three hub genes and the RS of each patient in the prognostic model was calculated using the following formula: RS = (1.167391 × expression of PPBP) + (0.900831 × expression of SEMA6B) + (0.471683 × expression of GCGR), thereby dividing the patients into low- and high-RS groups according to their median value of RS. The heatmap for THCA tissues in the combined, test, and training three sets (Figures 5A–C) revealed that expression levels of three prognostic hub genes were downregulated in the low-RS group. RS distribution (Figures 5D–F) and RS-related survival status among patients (Figures 5G–I) indicated that higher RS corresponded to higher mortality risk in THCA patients.

**TABLE 1:** Cox regression analysis for screening of IRDEGs influencing the THCA patients’ prognosis.

Gene ID	Univariate cox analysis		Multivariate cox analysis	
	HR (95%CI)	p-value	HR (95%CI)	p-value
PPBP	2.49 (1.50,4.13)	4.18E-04	3.21 (1.78,5.79)	1.02E-04
RBP4	3.20 (1.76,5.84)	1.42E-04		
SEMA6B	2.61 (1.49,4.62)	8.76E-04	2.46 (1.01,6.00)	4.74E-02
VGF	1.56 (1.22,2.00)	3.94E-04		
GCGR	1.75 (1.29,2.39)	3.72E-04	1.60 (1.05,2.44)	2.71E-02

IRDEGs, immune-related differentially expressed genes; THCA, thyroid carcinoma.

**TABLE 2:** Clinical characteristics between test and training cohorts.

Clinical characteristics		Number	Test (%)	Training (%)	*p*-value
Age	≤65	429 (85.97)	137 (83.54)	292 (87.16)	0.3376
	>65	70 (14.03)	27 (16.46)	43 (12.84)	
Gender	FEMALE	364 (72.95)	111 (67.68)	253 (75.52)	0.0811
	MALE	135 (27.05)	53 (32.32)	82 (24.48)	
Stage	Stage I-II	332 (66.53)	100 (60.98)	232 (69.25)	0.0889
	Stage III-IV	165 (33.07)	63 (38.41)	102 (30.45)	
	unknow	2 (0.4)	1 (0.61)	1 (0.3)	
T	T1-2	305 (61.12)	99 (60.37)	206 (61.49)	0.8227
	T3-4	192 (38.48)	65 (39.63)	127 (37.91)	
	TX	2 (0.4)	0 (0)	2 (0.6)	
M	M0	282 (56.51)	93 (56.71)	189 (56.42)	1
	M1	9 (1.8)	3 (1.83)	6 (1.79)	
	MX	208 (41.68)	68 (41.46)	140 (41.79)	
N	N0	229 (45.89)	70 (42.68)	159 (47.46)	0.2707
	N1	220 (44.09)	79 (48.17)	141 (42.09)	
	NX	50 (10.02)	15 (9.15)	35 (10.45)	

TX, unknown T stage; MX, unknown M stage; NX, unknown N stage.

**FIGURE 6:**
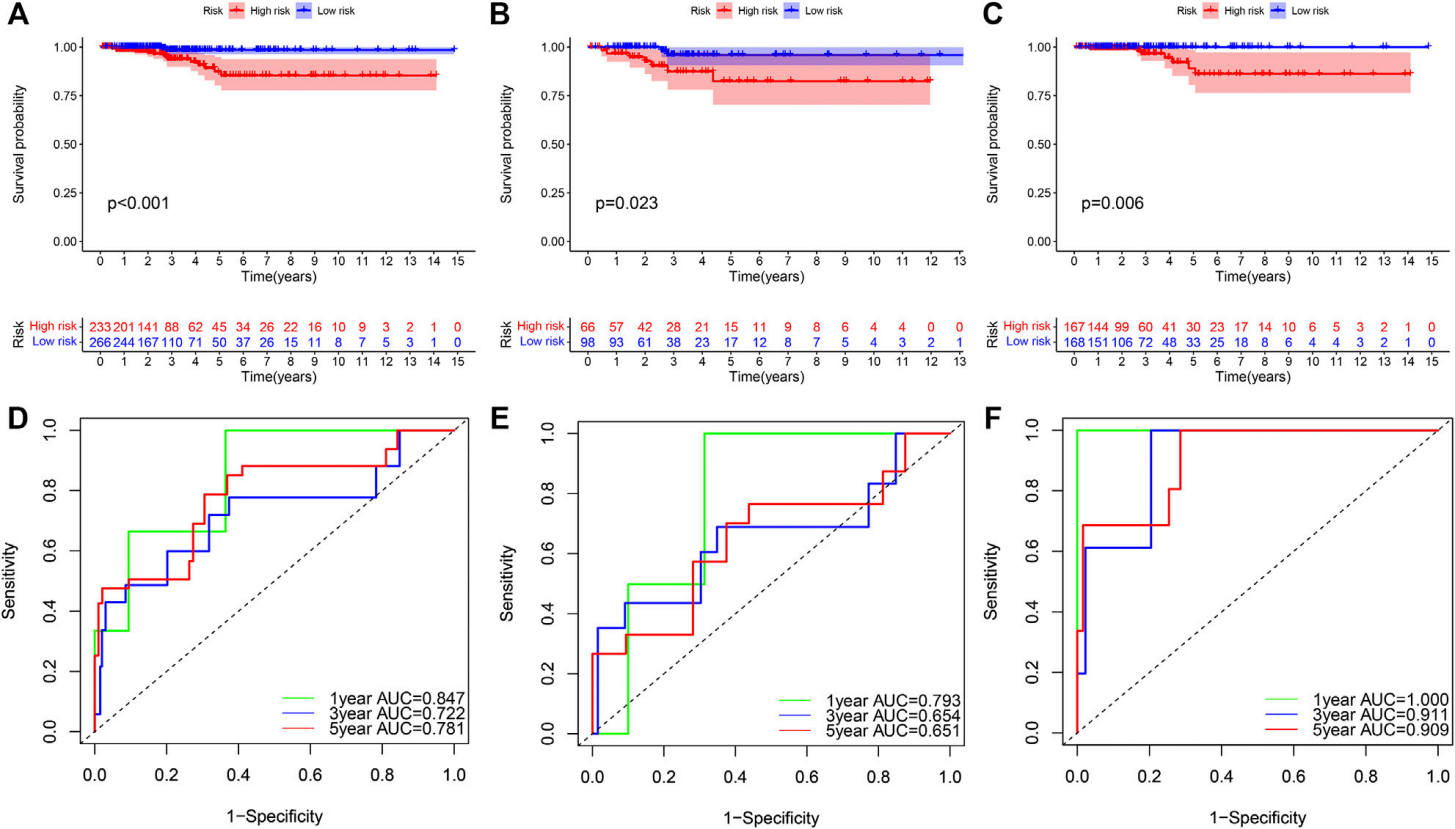
Cross-validation of a prognostic model in THCA. **(A–C)** OS analysis of THCA patients with high- and low-RS in the combined test, and training cohorts, with *p* < 0.001, *p* = 0.023, and *p* = 0.006, respectively. **(D–F)** ROC curve analysis for 1−, 3−, 5-years validation of RS model in three different cohorts. THCA, thyroid carcinoma; OS, overall survival; RS, risk score; ROC, receiver operating characteristic.

To further validate the reliability of THCA prognostic model, we further performed survival and ROC curves analyses. Kaplan–Meier survival plots for THCA patients in the combined, test and training three sets indicated that patients in the high-RS group had a substantially worse survival outcome than those in the low-RS group (Figures 6A–C), with *p* < 0.001, *p* = 0.023, and *p* = 0.006, respectively. Besides, the combined set showed that area under the curve (AUC) values for 1-, 3-, 5-yearsurvival were 0.847, 0.722, and 0.781, respectively (Figure 6D). Similarly, each AUC for 1-, 3-, 5-years survival was more than 0.6 in the test and training sets (Figures 6E, F). In brief, these findings demonstrated predictive accuracy and stability of the constructed model.

### Identification of Independent Risk Factors for Prognosis in THCA Patients

**FIGURE 7:**
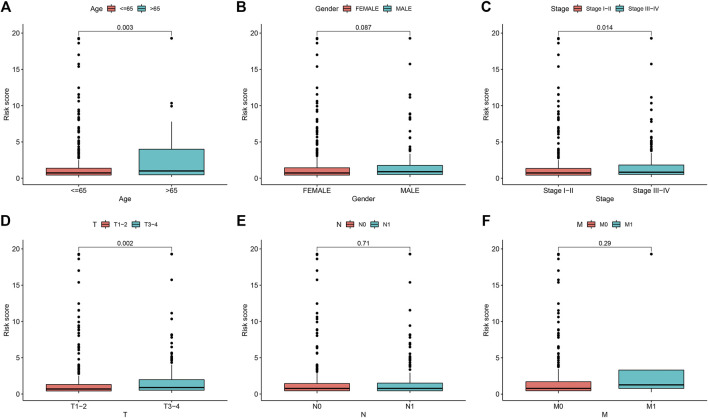
Correlation between RS and clinical characteristics in THCA. **(A)** age > 65 years old and < 65 years old **(B)** female and male **(C)** Stage I-II and stage III−IV, **(D)** T stage **(E)** Nstage **(F)** Mstage. Age over 65 years old, higher pathological stage, and higher T stage correlated with higher RS, with *p* = 0.003, *p* = 0.014, and *p* = 0.002, respectively. RS, risk score; THCA, thyroid carcinoma.

We conducted univariate and multivariate Cox regression analyses to determine the correlation between RS and clinical features in THCA patients. The results revealed that prognosis of THCA patients was linked to three significant risk factors, including the age at diagnosis, pathological stage and RS (Table 3). Boxplots of the relationship between patients’ clinical characteristics and RS revealed that patients aged over 65 years old (Figure 7A), had advanced pathological stages (Figure 7C), or had a higher T stage (Figure 7D) showed a higher RS, with *p* = 0.003, *p* = 0.014, and *p* = 0.002, respectively. However, no significant difference was observed in the correlations of RS with gender (Figure 7B), N (Figure 7E) and M (Figure 7F)stages, with *p >* 0.05. These results revealed that RS was associated with progression and development of THCA patients.

**TABLE 3:** Cox regression analysis for clinical characteristics and RS influencing prognosis in THCA patients.

Variable	Univariate cox analysis		Multivariate cox analysis	
	HR (95%CI)	*p*-value	HR (95%CI)	*p*-value
Age	1.16 (1.10,1.22)	2.27E-08	1.16 (1.09,1.22)	2.89E-07
Gender	1.92 (0.69,5.30)	2.09E-01		
Stage	2.42 (1.53,3.80)	1.39E-04	1.32 (0.74,2.37)	3.38E-01
Risk Score	1.01 (1.00,1.01)	5.88E-06	1.00 (1.00,1.01)	3.22E-03

RS, risk score; THCA, thyroid carcinoma.

### Associations of TMB With Prognosis and RS

**FIGURE 8:**
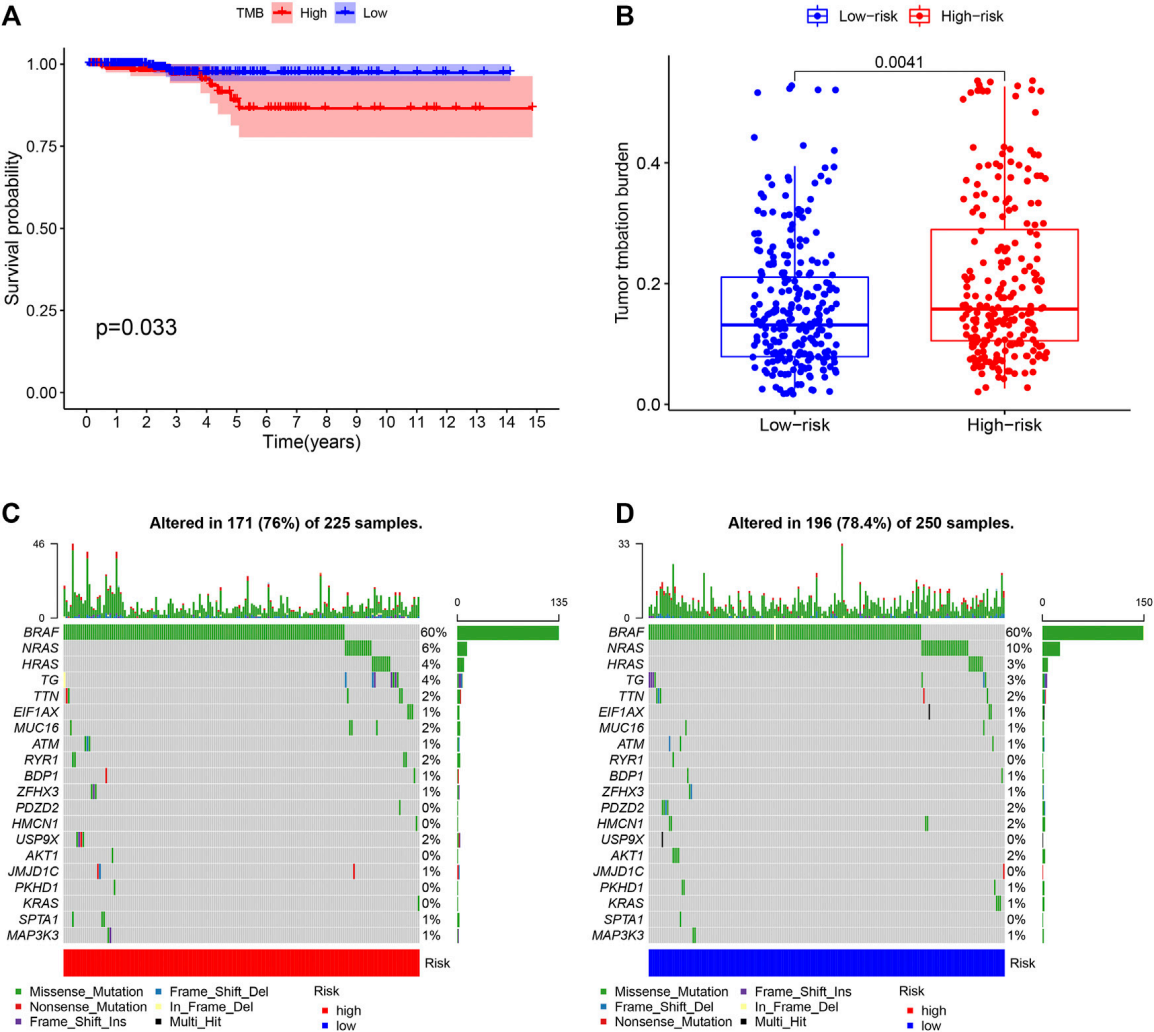
Association of TMB with OS and RS **(A)** Survival analysis of THCA patients with high- and low-TMB **(B)** Comparisons of TMB in low- and high-RS groups **(C) (D)** Waterfall plot for mutation profiles of the top 20 genes in THCA samples of high- and low-RS groups, respectively. Annotations with different colors at the bottom referred to the various mutation types and bar chart above presented mutation burden. The right showed name of mutated genes and the right displayed percent of gene mutation. TMB, tumor mutation burden; OS, overall survival; RS, risk score.

To examine the value of TMB in THCA prognosis, we analyzed the associations between TMB and overall survival (OS) time or RS. The Kaplan–Meier survival plot revealed that THCA patients with high TMB exhibited shorter OS time than those with low TMB (Figure 8A, *p* = 0.033). Besides, THCA patients in the high-RS group presented higher TMB levels than those in the low-RS group (Figure 8B, *p* = 0.0041). Such results indicated a negative relationship between THCA prognosis model and TMB. Waterfall plots displayed a landscape of the top 20 somatic gene mutations in 475 THCA samples from TCGA database, with different colors signifying different mutation types (Figures 8C,D).

### Evaluation of TICs and Immune Checkpoint Genes Expression

**FIGURE 9:**
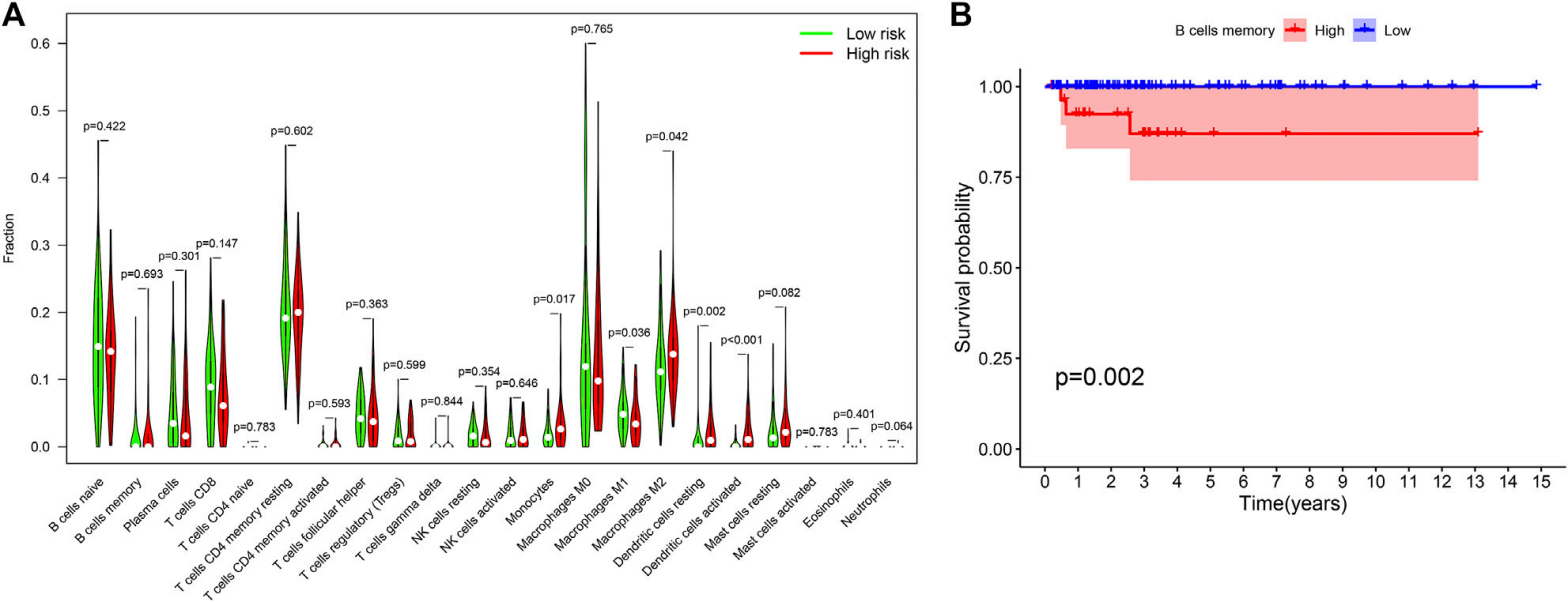
TICs profile of THCA cases **(A)** Violin plot for quantification of 22 TIC subtypes between low- and high- RS groups. **(B)** Memory B cells influencing survival outcome of THCA patients. TIC, tumor-infiltrating immune cell; THCA, thyroid carcinoma; RS, risk score.

To investigate the effect of constructed prognostic model on TICs we used CIBERSORT algorithm to calculate the distribution of 22 TIC subtypes in THCA samples. The Wilcoxon rank-sum test was utilized to compare the proportions of different TIC subtypes in high- and low-RS groups, and the findings were then visualized using a violin plot (Figure 9A). The results revealed that the relative abundance of monocytes, M2 macrophages, activated dendritic cells was significantly higher in high-RS group than those in low-RS group (*p* = 0.017, *p* = 0.042 and *p* < 0.001, respectively), whereas M1 macrophages and dendritic resting cells in high-RS group exhibited lower relative abundance (*p* = 0.036 and *p* = 0.002, respectively). According to their median TICs values, we classified THCA patients into high- and low- RS groups and then performed Kaplan–Meier survival analysis. The results displayed that high memory B cells was associated with poor OS (*p* = 0.002, Figure 9B).

**FIGURE 10:**
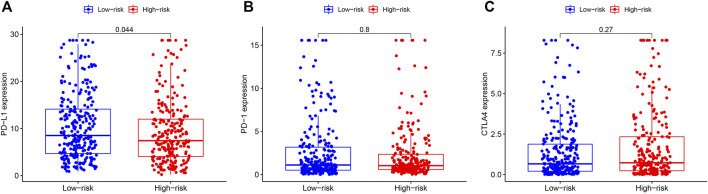
Expression levels of ICGs in high- and low-RS groups **(A)** PD-L1 expression of THCA patients in high- and low-RS groups. **(B)** PD-1 expression of THCA patients in high- and low-RS groups. **(C)** CTLA4 expression of THCA patients in high- and low-RS groups. RS, risk score; PD-L1, programmed cell death ligand 1; THCA, thyroid carcinoma; PD-1, programmed death 1; CTLA4, cytotoxic T lymphocyte-associated protein 4.

To further evaluate the effectiveness of immunotherapy on THCA, we analyzed expression levels of immunotherapy targets (PD-L1, PD-1 and CTLA4) in the high- and low-RS groups (Figure 10). The results revealed that the low-risk group significantly increased PD−L1 expression levels compared with the high-risk group (*p* = 0.044, Figure 10A), implying that low-RS THCA patients might be more susceptible to immunotherapy.

## Discussion

THCA is the fifth most prevalent cancer in the United States, with an annual incidence increase of ∼5% (Varricchi et al., 2019). Although most THCA patients have a favorable prognosis, approximately 15–20% of DTC patients, most ATC patients display resistance to standard treatment methods, such as RAI. Sorafenib and lenvatinib, two recently approved multikinase inhibitors (MKIs), have demonstrated encouraging outcomes in progressive, RAI-refractory DTC. However, adverse consequences have been identified, restricting their use (Cabanillas and Habra., 2016; Krajewska et al., 2017). Notably, the development of ICIs, such as anti–CTLA-4 and anti–PD-1 agents, have revolutionized THCA treatment (Antonelli et al., 2018; Varricchi et al., 2019). However, not all patients benefit from them due to individual variances. In the current study, we constructed a prognostic prediction model by screening IRDEGs to predict immunotherapy efficacy and survival outcome of THCA.

In the present study, we first identified 294 IRDEGs between THCA and normal samples and then explored potential functional enrichment pathways of these IRDEGs via KEGG and GO functional enrichment analyses. We discovered that the IRDEGs were primally enriched in some immune-related pathways, suggesting that IRDEGs might play a critical role in alteration of THCA immune microenvironment. Subsequently, three hub IRDEGs associated with THCA prognosis were identified, including PPBP, SEMA6B and GCGR. PPBP, alternatively known as C-X-C chemokine ligand 7, stimulates various cellular processes, such as DNA synthesis, glucose metabolism, intracellular cAMP accumulation and fibrinogen activator synthesis. Some studies have indicated that PPBP and its encoded proteins might be linked to the progression of Wilms tumor (Guo et al., 2017) and gastric cancer (Yamamoto et al., 2019). SEMA6B a member of the semaphorin family, is mainly involved in developing peripheral and central nervous systems (Andermatt et al., 2014). SEMA6B has been demonstrated to have a critical role in the prognosis of various tumors, such as gliomas (Sun et al., 2019), breast cancer (Murad et al., 2006) and testicular cancer (Ji et al., 2020). A recent study has shown that SEMA6B promoted occurrence and development of THCA via regulating Notch signaling pathway (Lv et al., 2021). GCGR, a member of G protein-coupled receptor family, is critical in regulating glucose homeostasis. Previous studies indicated that GCGR aberrant expression might lead to adverse survival in endometrial stromal sarcoma (Davidson et al., 2013), renal cell carcinoma (Schrödter et al., 2016), and non-small cell lung cancer (NSCLC) (Li et al., 2020). In addition, it has been revealed that GCGR overexpression results in poor survival of PTC by activating epithelial-mesenchymal transition (EMT) and P38/ERK pathway (Jiang et al., 2020). Although we speculated that these genes might be a potential therapeutic target and/or prognostic biomarker for treating THCA patients, this hypothesis still requires additional validation in future studies.

Next, we firstly built a THCA prognostic model using the three screened hub genes (PPBP, SEMA6B and GCGR), and validated the reliability of the prognostic model in THCA. The results in the combined, test, and training sets revealed that THCA patients in the high-RS group exhibited poor survival outcomes than those in the low-RS group, and AUC values were over 0.6, implying that the constructed prognostic model accurately predicted OS in THCA. Besides, we found that older patients in the more advanced stage had significantly greater RS levels than younger ones in the earlier stage, consistent with the conclusion drawn by Ibrahimpasic et al. (Ibrahimpasic et al., 2019).

TMB, as a novel biomarker, has been recently known to forecast the clinical efficacy of immunotherapy in many cancers (Samstein et al., 2019), since it is closely associated with tumor immune infiltration and microenvironment alteration (Kang et al., 2020). Zhou et al. (Zhou et al., 2021) discovered that patients in the low-TMB group exhibited a worse survival outcome and immune response than those in the high-TMB group in. However, few studies focused on the predictive value of TMB for immunotherapy in THCA patients. In this current study we revealed that THCA patients in the high-RS group possessed higher TMB than those in the low-RS group. Herein, we speculated that higher TMB might be correlated with a worse prognosis in THCA, consistent with the conclusion drawn by Zhang et al. (Zhang et al., 2019a) in their work on clear cell renal cell carcinoma. Besides considering the important roles of TICs in TME on prognosis of numerous malignancies (Zhang et al., 2003; Tran Janco et al., 2015), we further assessed the distribution of 22 TIC subtypes in THCA samples from high- and low-RS groups and the relationship with survival outcome. The analysis results revealed that patients in the high-RS group chiefly possessed a higher level of monocytes, M2 macrophages and activated dendritic cells (DCs). In contrast, those in the low-RS group exhibited higher proportions of M1 macrophages and resting DCs, manifesting that tumor-associated macrophages were associated with tumor progression (Jackaman et al., 2017). Nevertheless, the underlying mechanism remains yet unclear. Similarly, Travers et al. (Travers et al., 2019) found that increasing tumor-killing M1 macrophages and decreasing M2 macrophages in TME contributed to reduced TMB and improved survival in mice with ovarian cancer. Additionally, a study indicated a strong correlation between DCs and advanced patients with PTC (Bergdorf et al., 2019), which might explain why THCA samples in the high-RS group exhibited advanced pathological stages. Besides, it has been reported that tumor-infiltrating memory B cells are linked to superior clinical outcomes in breast cancer (Garaud et al., 2019). In contrast, in the present study, we revealed that a high percent of memory B cells was highly correlated with poor survival in THCA.

Immune checkpoint molecules, such as PD-L1, PD-1 and CTLA4, have been demonstrated to connect with the efficacy of immunotherapy (Kalbasi and Ribas., 2020) Hence, we further investigated these biomarkers, including PD-L1, PD-1 and CTLA4, expression levels of THCA patients between in the high- and low-RS group. Results showed that PD-L1 expression was significantly upregulated in the low-RS group compared to the high-RS group, suggesting that this prognostic model might have ability to determine THCA patients’ response to immunotherapy. Based on these findings, we supposed that high TMB and low PD-L1 expression in THCA patients might respond poorly to immunotherapy.

## Conclusions

In conclusion, this study constructed and validated a THCA prognostic prediction model based on TCGA database, displaying good predictability and reliability for THCA prognosis. To our knowledge, our group was the first to screen out three potential therapeutic target genes and elucidate the association of TICs with RS and OS in THCA. Additionally, we figured out that THCA patients in the high-RS group had high TMB and low PD-L1 expression, establishing a baseline and reference for predicting THCA immunotherapy efficacy in clinical trials. However, future investigations require relevant basic experiments and large sample clinical trials.

## Data Availability

The datasets presented in this study can be found in online repositories. The names of the repository/repositories and accession number(s) can be found in the article/supplementary material.
